# Trigeminal Herpes Zoster Transited to Ipsilateral Occipital Neuralgia

**DOI:** 10.3390/neurolint14020036

**Published:** 2022-05-18

**Authors:** Keita Takizawa, Zhimin Yan, Jumi Nakata, Andrew Young, Junad Khan, Mythili Kalladka, Noboru Noma

**Affiliations:** 1 Department of Oral Medicine, Nihon University School of Dentistry, Tokyo 101-8310, Japan; takizawa.keita@nihon-u.ac.jp; 2 Department of Oral Medicine, Peking University School and Hospital of Stomatology, Beijing 100871, China; yzhimin96@163.com; 3 Division of International Medicine, Towa Hospital, Tokyo 120-0003, Japan; jumi.nakata@gmail.com; 4 Department of Diagnostic Sciences, Arthur Dugoni School of Dentistry, University of the Pacific, San Francisco, CA 94103, USA; ayoung@pacific.edu; 5 Eastman Institute for Oral Health, University of Rochester Medical Center, 625 Elmwood Ave, Rochester, NY 14620, USA; junad_khan@urmc.rochester.edu (J.K.); dr.mythili@gmail.com (M.K.)

**Keywords:** occipital neuralgia, herpes zoster, varicella–zoster virus

## Abstract

The pain of occipital neuralgia (ON) is thought to be secondary to trauma or injury to the occipital nerve at any point along the course of the nerve. ON may also be caused by an infectious process (herpes zoster) or compression of the nerve. The patient, in this case, presented to our clinic with complaints of occipital pain and rash and swelling of the right lower jaw. One week before presenting to our clinic, the patient developed severe pain in the first division of the trigeminal region with erythema and vesicles. A blood test showed a remarkably high antibody titer for varicella–zoster virus (VZV). The patient was prescribed oral valacyclovir (Valtrex^®^) (3000 mg/day), which resulted in the complete remission of the rash and blisters in the occipital region. This highlights the importance of considering neuroanatomy of the trigeminal region and cervical nerve.

## 1. Introduction

Herpes zoster (HZ) results from reactivation of latent varicella–zoster virus (VZV) in the dorsal root ganglia, and VZV propagates along the sensory nerves to the skin, causing inflammatory lesions in the peripheral nerves and areas of skin [1]. Occipital neuralgia may reach the fronto-orbital area through trigeminocervical inter-neuronal connections in the trigeminal spinal nuclei [2].

To our knowledge, case reports regarding trigeminocervical inter-neuronal connections have not been reported. Herein, we report a case of herpes zoster in the first branch of the trigeminal nerve followed by occipital neuralgia with herpes zoster in the second cervical nerve area on the ipsilateral side. We also created a neuroanatomical schematic for the trigeminal spinal nucleus caudalis and upper cervical spinal cord and discussed the use of local anesthetic as a diagnostic aid.

## 2. Case

**Figure 1:**
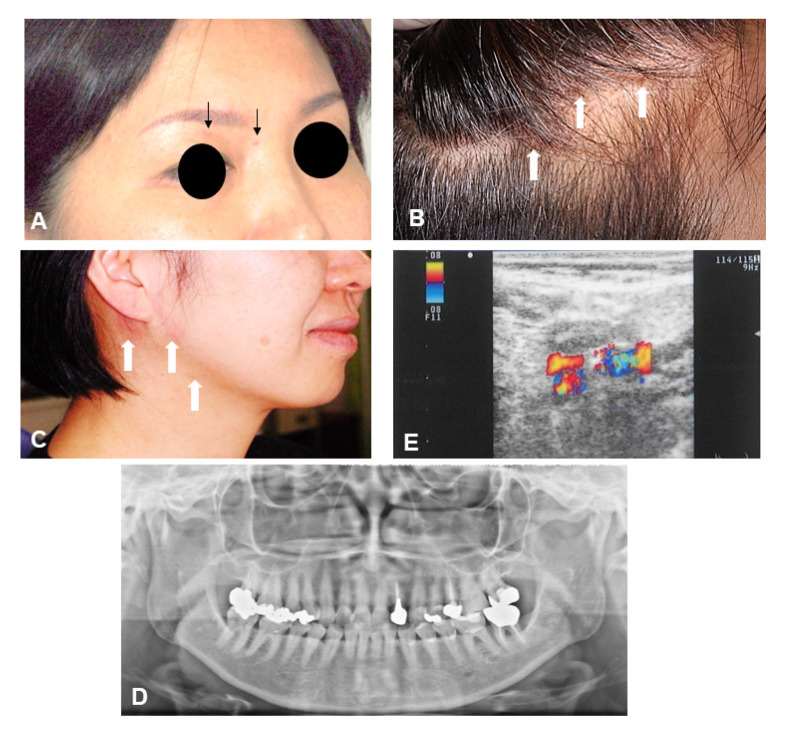
(**A**–**C**): The patient had a burning pain, edematous erythema and vesicles, and swelling around the eyelids and on the scalp after a period of days and consulted with a neurosurgeon. (**D**): Panoramic radiograph shows mild resorption of mandibular condyle on the right side. (**E**): Ultrasonography showed lymphadenitis.

A 47-year-old female presented to our clinic with complaints of occipital pain and swelling of the right lower jaw. Eighteen days before seeking the treatment, the patient complained of stabbing pain from the right temporal region to the parietal region. Approximately 10 days prior to the presentation, she had swelling in the right submandibular region and a rash on the occipital region. At her first visit to our clinic, crusts were observed on the parietal head region and upper eyelids, suggesting a rash of the first division of the trigeminal region that had almost healed (Figure 1A,B). The patient complained of tenderness over the right occipital region and allodynia during innocuous stimulation of the hair. Erythema and vesicles were found on the lower right jaw and retroauricular region (Figure 1C).

Examination of the temporomandibular joint revealed an active range of motion of >40 mm and no tenderness in the left masseter muscle. Cranial nerve examination revealed slight sensory loss to light touch in the left lower lip and tongue. A panoramic radiograph showed the right mandibular condyle was slightly flattened (Figure 1D).

Laboratory investigations revealed a hemoglobin level of 14.6 g/dL, red blood cell count of 466 million cells/mcL, white blood cell count of 5.75 K/mcL, erythrocyte sedimentation rate of 5 mm/h, platelet count of 24.5 K/mcL, MCV of 91.6 u3, MCH of 31.3 pg, and MCHC of 34.2%. Plasma units with titers of complement-fixing antibody to varicella–zoster virus (VZV) were greater than 16.

### Ultrasonography Findings

Ultrasonography showed mild overall swelling in the submandibular region. The internal nodal region was clear. Findings were suggestive of lymphadenitis (Figure 1E). The patient was prescribed oral valacyclovir (Valtrex^®^) 1000 mg three times daily for 7 days, which resulted in the complete remission of the pain, rash, and blisters.

## 3. Discussion

The trigeminal spinal tract nucleus spans from the caudal region of the pons to the upper cervical spinal cord and is anatomically continuous with the dorsal horn of the upper cervical spinal cord. This anatomical or functional connection is called the trigeminocervical complex (TCC) [3].

**Figure 2:**
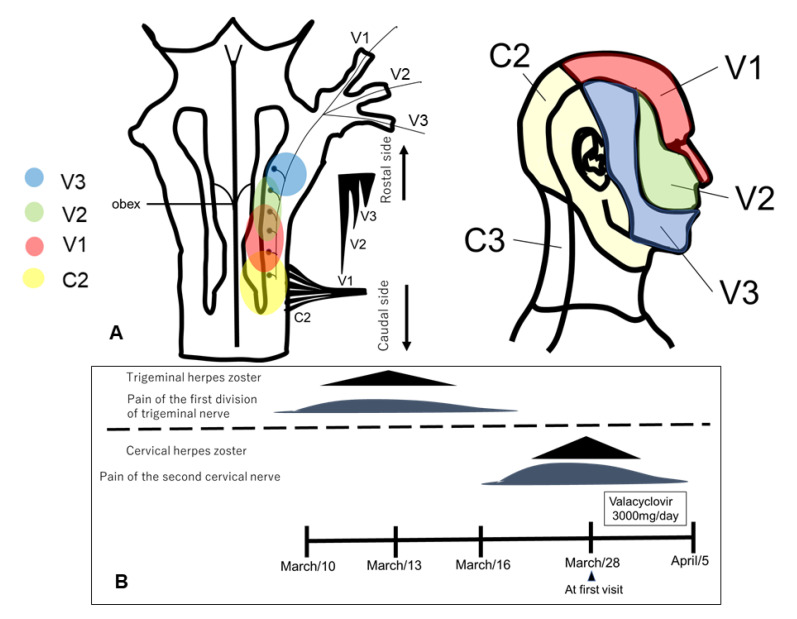
(**A**): Organization of trigeminal spinal nucleus caudalis and upper cervical spinal cord from peripheral region. V1–3: trigeminal regions 1–3. C2,3: cervical spinal cord. (**B**): Clinical course from 10 March to 5 April.

From the perspective of anatomic morphology, the first branch of the trigeminal afferent nerve descends toward the caudal side to the upper cervical spinal cord and is densely distributed in the trigeminal spinal tract nucleus, but the majority of the second and third branches of the trigeminal nerve are located rostral to the upper cervical spinal cord (Figure 2A) [3].

In animal experiments, it has been reported that stimulation of the greater occipital nerve region causes central sensitization of the first branch of the trigeminal nerve [4]. In an immunohistologic study, Goadsby et al. demonstrated that electrical stimulation of the first branch of the trigeminal nerve increased the expression level of c-fos in the dorsal horn of the upper cervical spinal cord C2 [5]. Since the distribution of lesions, in this case, was in the lesser occipital nerve that originated from the cervical nerve C2 and the first branch of the trigeminal nerve on the ipsilateral side, we hypothesized that the TCC was involved in the spread of neuroinflammation caused by VZV.

Temporomandibular disorder (TMD) is often prominent in the temporal and preauricular regions [6]. The differential diagnosis for occipital pain includes TMD, migraine, cluster headaches, and the various diseases of the cervical spine, especially those involving C2–3 nerve roots. The presence of myofascial trigger points in the posterior cervical muscles has been shown to mimic the pain of ON. Therefore, all patients presenting with signs and symptoms of ON should be examined for myofascial trigger points.

A local anesthetic block of the suspected nerve may aid the diagnostic process [7] but requires a thorough familiarity with the local anatomy. Relief from an occipital nerve block is also not entirely specific for ON, and false-positive results occur with TMD, migraine, and cluster headaches. According to a previous review article, the occipital nerve block may be a good treatment option for ON, although we did not perform a diagnostic block on the patient [8].

## 4. Conclusions

This case demonstrated the relevance of the neuroanatomical connection between the trigeminal nerve and the upper cervical spinal cord. A thorough understanding of neuroanatomy would allow for anesthetic nerve blocking as a diagnostic aid, though great care should be taken when doing so, and the potential for a false positive should be considered.

## Data Availability

Not applicable.
